# Target search by an imported conjugative DNA element for a unique integration site along a bacterial chromosome during horizontal gene transfer

**DOI:** 10.1093/nar/gkad068

**Published:** 2023-02-10

**Authors:** Rinat Arbel-Goren, Saria A McKeithen-Mead, Dominik Voglmaier, Idana Afremov, Gianluca Teza, Alan D Grossman, Joel Stavans

**Affiliations:** Department of Physics of Complex Systems, Weizmann Institute of Science, Rehovot 76100, Israel; Department of Biology Massachusetts Institute of Technology, Cambridge, MA 02139, USA; Department of Physics of Complex Systems, Weizmann Institute of Science, Rehovot 76100, Israel; Department of Physics of Complex Systems, Weizmann Institute of Science, Rehovot 76100, Israel; Department of Physics of Complex Systems, Weizmann Institute of Science, Rehovot 76100, Israel; Department of Biology Massachusetts Institute of Technology, Cambridge, MA 02139, USA; Department of Physics of Complex Systems, Weizmann Institute of Science, Rehovot 76100, Israel

## Abstract

Integrative and conjugative elements (ICEs) are mobile genetic elements that can transfer by conjugation to recipient cells. Some ICEs integrate into a unique site in the genome of their hosts. We studied quantitatively the process by which an ICE searches for its unique integration site in the *Bacillus subtilis* chromosome. We followed the motion of both ICE*Bs1* and the chromosomal integration site in real time within individual cells. ICE*Bs1* exhibited a wide spectrum of dynamical behaviors, ranging from rapid sub-diffusive displacements crisscrossing the cell, to kinetically trapped states. The chromosomal integration site moved sub-diffusively and exhibited pronounced dynamical asymmetry between longitudinal and transversal motions, highlighting the role of chromosomal structure and the heterogeneity of the bacterial interior in the search. The successful search for and subsequent recombination into the integration site is a key step in the acquisition of integrating mobile genetic elements. Our findings provide new insights into intracellular transport processes involving large DNA molecules.

## INTRODUCTION

Conjugation is a horizontal gene transfer process that contributes significantly to the generation of genetic diversity in bacteria (1). During conjugation, or mating, DNA is transferred directly from a donor bacterial cell to a recipient. There are two types of conjugative elements, plasmids and integrative and conjugative elements (ICEs). Whereas plasmids are extrachromosomal, ICEs reside integrated in the chromosome of a host cell. As a potent force of bacterial evolution, conjugation has played a fundamental role in organizing the genetic information on chromosomes, distributing genes involved in processes such as pathogenesis, antibiotic resistance and metabolism (2,3).

Although stably maintained integrated in the host chromosome, ICEs can excise from the chromosome to form a circular DNA that can then transfer from a donor cell to a recipient where it is first extrachromosomal but then integrates into the chromosome of the new host to form a stable transconjugant. Many ICEs integrate into a specific, often unique site in the bacterial chromosome. Locating and integrating into the proper site is an essential step in the lifecycle of these widespread elements, yet nothing is known about how an ICE locates the proper integration site.

ICE*Bs1* is a relatively small ICE (∼20 kb) found in the chromosome of *Bacillus subtilis* (4,5). It integrates into a preferred attachment site, *attB*, located in a tRNA gene (*trnS-leu2*). In the absence of its normal attachment (integration) site, ICE*Bs1* can integrate into secondary sites with reduced frequency, and integration into these secondary sites is detrimental to both the element and its bacterial host (6), highlighting the importance of locating the proper site for integration.

The search for *attB* by ICE*Bs1* belongs to a general class of search problems that are ubiquitous in many biological contexts, ranging from the search for cognate sites by transcription factors (7,8), the repair of DNA lesions (9), to horizontal gene transfer processes other than conjugation (10) and the RNA-guided search of specific sequences by CRISPR (11,12). Common to all these processes is the search for a short ∼10–20 bp-long target site along a ∼1 to >5 Mb-long chromosome, within the complex and crowded bacterial cytoplasm (13). Several investigations have provided evidence supporting the notion that the cytoplasm is a non-equilibrium active medium in which fluctuations are primarily metabolic, driven by ATP and GTP usage (14,15). The *in vivo* search mechanisms allowing an ICE to locate its integration site in a transconjugant are largely unknown.

Here, we focus on the quantitative characterization of the *in vivo* dynamics of both ICE*Bs1* and its integration site *attB* during the search process leading to integration into the chromosome of a transconjugant. We visualize both loci within the same cell in real time by labelling each locus with multiple binding sites for a fluorescent reporter protein, specifically TetR-mApple2 (*tetO* sites inserted close to the *attB* site) or LacI-GFP (*lacO* sites inserted at the ICE*Bs1*), as represented in Supplementary Figure S1 (16). Previous investigations in *Escherichia coli* revealed that the motion of chromosomal loci is subdiffusive and consistent with fractional Brownian motion (15,17). Moreover, anisotropy between the longitudinal and transversal motions of chromosomal loci has been reported for *E. coli* (18) and *Vibrio cholerae* (19), possibly reflecting an underlying structural anisotropy in the bacterial chromosome. Our experimental findings indicate that far from being diffusive, the search is a non-equilibrium, metabolically driven process in which the rapid, sub-diffusive motion of ICE*Bs1* across the whole cell is punctuated by episodic explorations of local chromosomal regions, until the asymmetrically moving target site (*attB*) is found and integration takes place. Our results underscore the importance of chromosomal structure in the search, the efficiency of target location within a crowded environment, and heterogeneity as an essential principle in bacterial subcellular behavior.

## MATERIALS AND METHODS

### Media and growth conditions

*Bacillus subtilis* strains were grown in LB medium for routine cloning and strain constructions. Strains for experiments were grown in defined S7 minimal salts medium (containing 50 mM MOPS [morpholinepropanesulfonic acid]) supplemented with l-arabinose (1%), phenylalanine (40 μg/ml), tryptophan (40 μg/ml), threonine (200 μg/ml) and methionine (40 μg/ml), as needed. Xylose (1%) was added to induce expression from Pxyl-*rapI* (all reagents were from Sigma unless specified).

### Live-cell imaging and mating conditions

Donors and recipients were colony purified from frozen (−80°C) stocks on LB agar plates with the appropriate antibiotics overnight at 42°C. Cells from a single colony were inoculated into liquid LB medium and grown to an optical density OD_600_ of ∼0.8 to 1. Cells were then diluted into defined minimal medium with arabinose as the carbon source to an OD_600_ of ∼0.02. After at least three to four generations (OD_600_ of ∼0.2), expression of *rapI* from P*xyl*-*rapI* was induced by addition of xylose to the donors. Cells were grown for another hour, to allow for ICE*Bs1* gene expression and excision, and then washed three times. Donors and recipients were then mixed at a ratio of ∼1 donor per three recipients at a concentration of ∼10^8^ cells/ml. Five microliters of this mating mix were placed on an agarose pad (low melting 2% agarose dissolved in defined minimal growth medium). The pad was circular with a volume of ∼100 μl and a height of ∼2 mm. Then the pad was laid on top of a MatTek device that has coverslip glass (#1.5) built into a 35 mm dish. The effect of different treatments was studied by addition of 5 μl of 100× stock solutions on top of the agar pad, at 37°C and then tracking cells using fluorescence microscopy. The 100× concentrations of compounds and total times of cell tracking were: Chloramphenicol (50 mg/ml, 30 min); rifampicin (10 mg/ml, 15 min) and 2,4-dinitrophenol (DNP) (45 mg/ml, 30 min). Time-lapse images were recorded typically between 1–3 h following mating.

### Bacterial strains and growth

The *B. subtilis* strains used are listed in Table 1. All are derivatives of JH642 (20,21). Strains were constructed by standard procedures using natural transformation. New alleles were constructed according to the following and transformed into a clean JH642 or ICE*Bs1* null JH642 derived strain (JMA222) (22). Alleles were transformed by natural transformation as described into appropriate backgrounds. *tetR-mApple* was constructed by replacing the *yfp* coding sequence in pPSL38 (23) with *mApple* (24) to produce pSAM021. pSAM021 was integrated into the chromosome at *cgeD*. Diagnostic PCR was used to verify integration by double-crossover. Δ*yddS*::*tetO240* is linked to *attB* and was constructed by inserting the *tetO* array from pLAU44 (25) by restriction digest cloning into the plasmid pSAM221. pSAM221 was constructed by amplifying two fragments from JH642 genomic DNA and the chloramphenicol resistance cassette from pGEMcat (26). Fragments were inserted into digested pUC19 by isothermal assembly reaction generating pSAM308 (27). The plasmid pSAM308 was integrated into the chromosome by double-crossover into *yddS* and confirmed by diagnostic PCR.

**Table 1.** *B. subtilis* strains used

Strain	Relevant genotype^a^ (comments)	Fluorescence
SAM318 (recipient)	ICE*Bs1*^0^ (cured of ICE*Bs1*) *ΔyddS*::(*tetO240 cat*; near *attB*) *ΔcgeD*::{Ppen(mutTAGG)-*tetR*-*mApple2 tet*} *ΔamyE*::(Ppen-*lacI*-*gfpmut2 spc*)	GFP (foci if ICE*Bs1*^+^); mApple2 (*attB*)
MMB1277 (donor)	ICE*Bs1 Δ*(*rapI-yddM)*::{*lacO* (arrays in ICE*Bs1*) *kan*} *amyE*::(Pxyl-*rapI spc*)	None
SAM837	ICE*Bs1 Δ*(*rapI-yddM)*::{*lacO* (arrays in ICE*Bs1*) *kan*} *ΔyddS*::(*tetO240 cat*; near *attB*) *ΔcgeD*::{*tet* Ppen(mutTAGG)-*tetR*-*mApple2*} *ΔamyE*::(Ppen-*lacI*-*gfpmut2 spc*)	GFP (ICE*Bs1*); mApple2 (*attB*)
SAM049	*ΔcgeD*::{*tet* Ppen(mutTAGG)-*tetR*-*mApple2*} 300°::(*tetO kan*)	mApple2 (300°)

^a^All strains contain the *trpC2 pheA1* alleles (not indicated).

### Microscopy and image acquisition

The setup consists of an inverted microscope (Nikon, Ti2-Eclipse) with a motorized x–y-stage (Prior). Stage, sample holder and objective (100×/1.45 oil, Nikon) are inside a temperature box (Okolab) at 37°C. Phase contrast and fluorescence images were acquired with a sensitive camera (Andor DU-987), using the NIS-Element Advanced Research software (Nikon, version 5.02.01). An experiment consists of a sequence of images taken from a sample. An image consist of 512 × 512 pixels (px) with a resolution of 109.76 nm/pixel. Images were typically acquired every about 4 s or 30 s over a total time of around 6 min or 45 min. These parameters allowed us to capture essential features of the dynamics while minimizing photo-bleaching. Cells were illuminated with light of the appropriate wavelength, produced by a light emitting diode set (CoolLED pE-4000), using appropriate filter sets for each fluorescent reporter: GFP (Chroma, 49002- ET-GFP (FITC/Cy2)) and RFP (Chroma, 49008-ET-mCherry, Texas Red).

### Image analysis

Only transconjugants of strain SAM318 that displayed two *attB* loci and one ICE*Bs1* were analysed. In the strain in which ICE*Bs1* was fully integrated (SAM837), two *attB* foci and two ICE*Bs1* foci were analysed in each cell. The presence of two foci indicates that that region of the chromosome has already been replicated. All image processing and data analysis were carried out using a MATLAB (MathWorks) algorithm developed in our laboratory. The code aims at finding the trajectories of ICEBs1 and chromosomal attachment sites labeled with fluorophores and characterize their dynamics. NIS-element images were converted to TIFF files and then individual cell segmentation was carried out in phase contrast images to determine the cell boundaries. The algorithm then calculates the center of mass of each cell and its centerline.

ICE*Bs1* and *attB* positions were then determined with sub-pixel resolution by fitting a Gaussian plus a constant to pixel clusters above an appropriate threshold within the cell boundaries. Their positions were then determined with respect to the cells’ center of mass, with $x( t )$ being the distance of the locus to the centreline (transversal direction) and $y( t )$ being the position along the centreline relative to the center of mass (longitudinal direction).

## RESULTS

### Search and location of the integration site *attB* by ICE*Bs1* in individual cells

**Figure 1.**
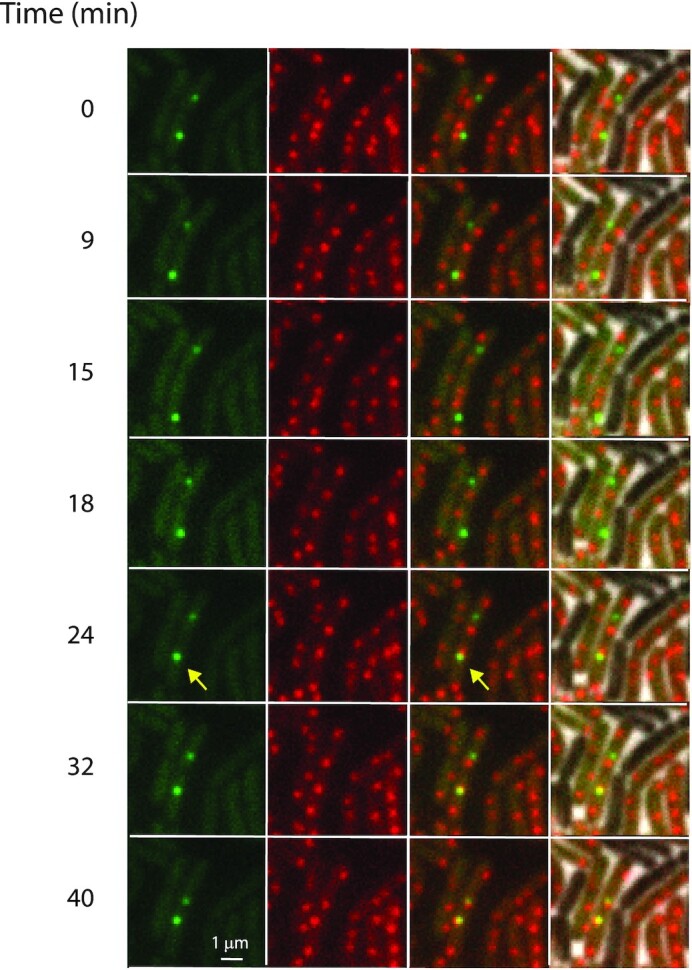
Search for the chromosomal integration site *attB* by ICE*Bs1*. First column: snapshots of ICE*Bs1* labelled with GFP in transconjugant cells at the indicated times (green dots), ∼1 h after mating of MMB1277 as a donor and SAM318 as a recipient (Materials and Methods), with $t\ = \ 0$ corresponding to the time at which the first image was recorded; second column: *attB* labelled with mApple2 (red dots); third column: overlay of GFP (ICE*Bs1*) and mApple2 (*attB*) images; fourth column: overlay of third column with phase contrast images, showing the presence of non-fluorescent donor cells. Arrows show a moving ICE*Bs1* that integrates (see also Movie S1). The scale bar represents 1 μm.

We used fluorescence reporters and microscopy (28), to visualize the appearance of transconjugants after mating donor and recipient strains. Typically, transconjugants could be visualized about one hour following mixing donors and recipients (Figure 1) and the positions of ICE*Bs1* and *attB* loci were followed ∼2–4 generations after mixing donors and recipients. The percentage of transconjugants was ∼5%, a mating efficiency that is comparable to previous observations (16).

**Figure 2.**
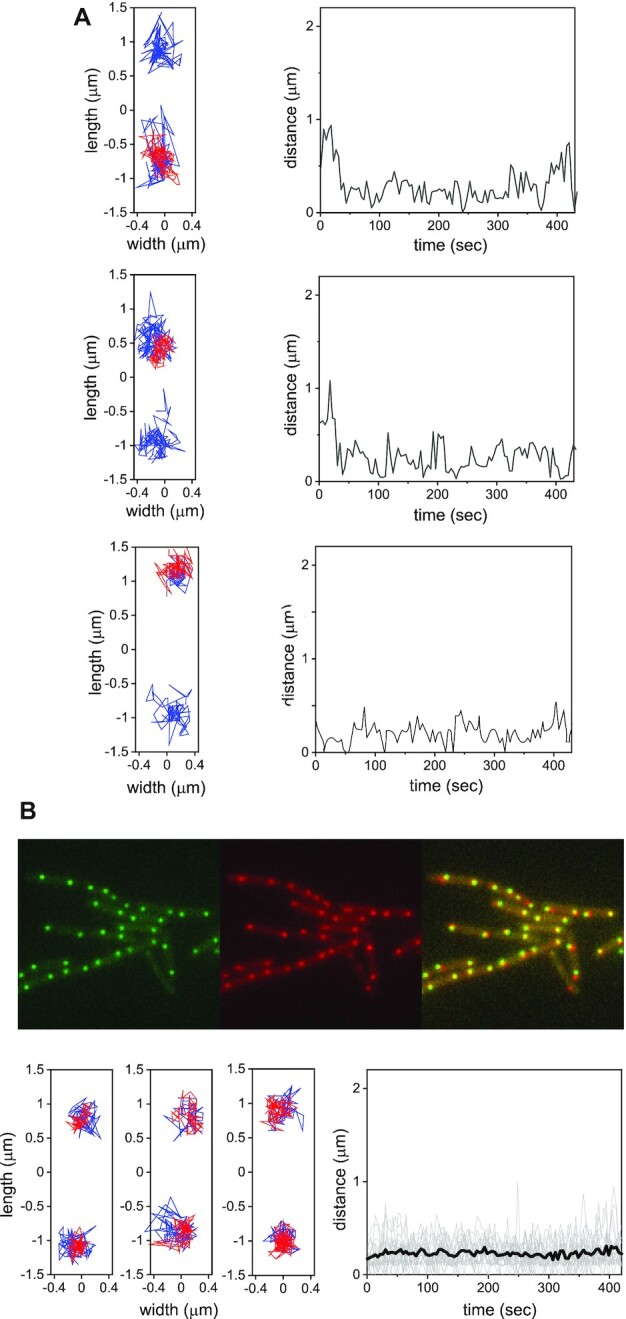
Trajectories of integrated ICE*Bs1* in transconjugants. (**A**) Three representative events of integrated ICE*Bs1*. The left panels show two-dimensional trajectories of *attB* (blue) and ICE*Bs1* (red) in transconjugants; the mean interval between successive positions in the trajectories is ∼4 s. Right panels: instantaneous distance between ICE*Bs1* and the nearest *attB* as a function of time. (**B**) Top: fluorescence images of integrated ICE*Bs1* (green), *attB* (red) and the overlay in strain SAM837 in which ICE*Bs1* is integrated and cannot excise. Bottom: left panels show representative two-dimensional trajectories of *attB* and ICE*Bs1* in strain SAM837. The right panel shows the instantaneous distance between ICE*Bs1* and the nearest *attB* as a function of time for 20 pairs (grey lines) and their mean (black line).

**Figure 3.**
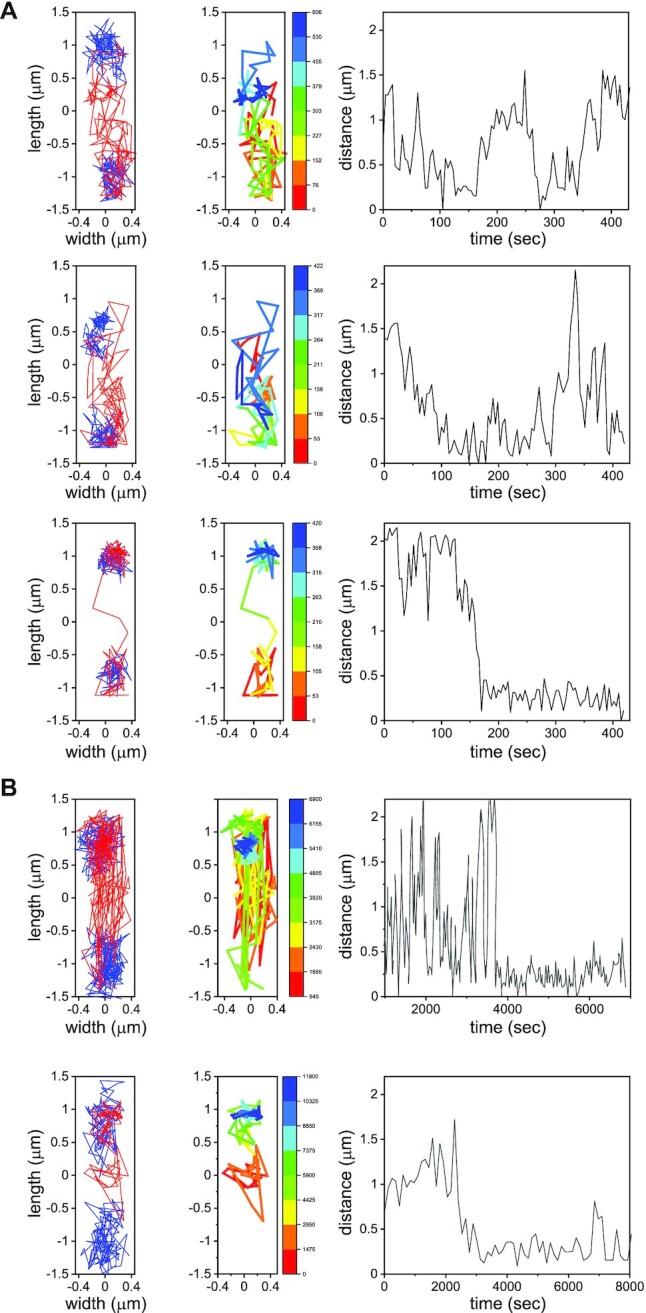
Target search for *attB* by ICE*Bs1*. (**A**) First panels from left to right: Representative two-dimensional trajectories of *attB* (blue) and ICE*Bs1* (red) in three transconjugants; the interval between successive positions in the trajectories is ∼4 s. Second panel: ICE*Bs1* trajectories color-coded according to time. Third panel: distance between ICE*Bs1* and one of the *attB* sites (there are two because most cells have a partly replicated chromosome). (**B**) As in (A), for typical intervals of ∼120 s between frames.

**Figure 4.**
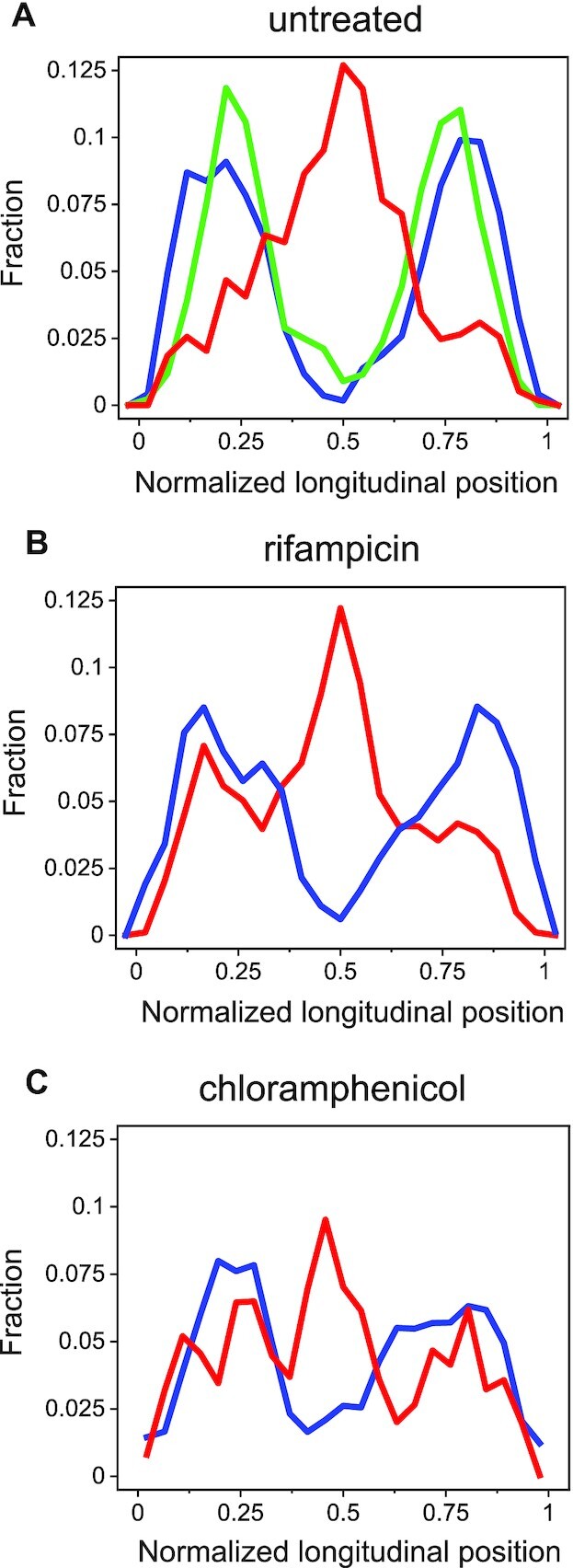
Position distributions of chromosomal loci and ICE*Bs1* along the cell's main axis. (**A**) Distributions of normalized longitudinal positions of a *tet* array in *yddS* (∼47°; 13.16 centisomes) near *att*B (blue, ∼45°; 12.56 centisomes, *n* = 8040), Supplementary Figure S1) and a *tet* array in a different chromosomal locus (green, 300°, using SAM049, *n* = 10 560), and ICE*Bs1* (red, *n* = 1134) along a cell's main axis. Only ICE*Bs1* trajectories whose displacements were >200 nm were included. Longitudinal positions were normalized by the cell length. (**B**) Same as in (A), but for cells following treatment with rifampicin (red *n* = 933 and blue *n* = 3196). (**C**) Same as in A, but for cells following treatment with chloramphenicol (red *n* = 7646 and blue *n* = 5270).

To capture the dynamical characteristics of the search process in the transconjugants, we sampled the motion of ICE*Bs1* and *attB* in transconjugant cells at various temporal resolutions (Video 1 illustrates the search and eventual location of an *attB* locus at a low temporal resolution of 2 min/frame). Two-dimensional trajectories of *attB* and ICE*Bs1* in representative cells are shown (Figures 2 and 3) soon after detection of ICE*Bs1*. The behavior of *attB* trajectories was generally uniform, with two loci (indicative of a partly replicated chromosome) found typically at ¼ and ¾ along the longitudinal axis (Figure 4A). These positions are in accordance with previous observations (29).

In contrast, ICE*Bs1*s exhibited substantial dynamical heterogeneity: In some cells there was considerable movement of ICE*Bs1*, quite different from that of *attB*. In the majority of cells, the trajectories of ICE*Bs1* overlapped considerably with those of *attB* (Figure 2A). Cells in which ICE*Bs1* and *attB* trajectories were similar had likely integrated the element into the chromosome (into *attB*). Plots of the two-dimensional Euclidean distance between ICE*Bs1* and *attB* trajectories with which they overlap are shown in Figure 2A. Note that the distance between fluorescence spots fluctuates around ∼200 nm. To support the notion that such events indeed correspond to integration, we measured the distance between *attB* and ICE*Bs1* loci in a strain (SAM837) bearing integrated ICE*Bs1* that was unable to excise (Figure 2B). The average distance between spots in this strain is indeed ∼200 nm. We surmise that the distance between tandem operator sites labelling *attB* and ICE*Bs1*s (∼15 kb, Supplementary Figure S1), together with local chromosome de-compaction due to both TetR-mApple2 and LacI-GFP polymerization on the respective tandem operator sites, account for this distance.

About 45 ± 2% (*n* = 1223) of the total number of transconjugants, displayed mobile behavior without being integrated. These included 14 ± 2% (mean ± SE, *n* = 868, five independent experiments) that displayed fast motion of ICE*Bs1* that often spanned a large portion of the cell volume. This fast motion was punctuated by episodes of more compact exploration, until eventual integration at *attB* (Figure 3). A small group of cells, had ICE*Bs1* that was kinetically trapped, and the extent of motion of ICE*Bs1* was significantly reduced (Supplementary Figure S2). Pooling together the longitudinal positions of ICE*Bs1* that displayed large-scale motion with steps >200 nm (to eliminate elements that had integrated), we calculated the spatial distribution of ICE*Bs1* along the main cell axis. We found that the distribution of freely moving ICE*Bs1* peaks at the cell center, and overlaps moderately with the longitudinal distribution of *attB* loci, allowing ICE*Bs1* to locate *attB* (Figure 4A). Note that inhomogeneous molecular distributions in bacterial cells have been reported previously (30). To gain further insights into the mechanisms of search, we characterized quantitatively the motion of each locus separately.

### The motion of the target chromosomal integration site *attB* is anisotropic

**Figure 5.**
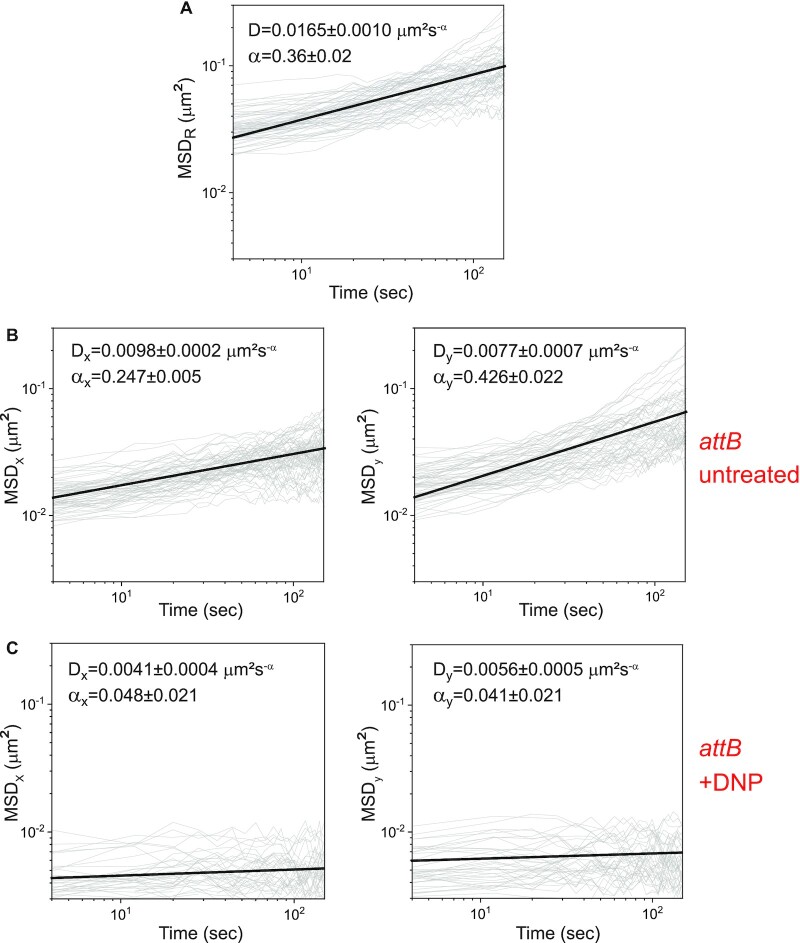
Anisotropic mean square displacement of *attB*. (**A**) Time-averaged mean square displacement $MS{D}_R\,\,$of individual two-dimensional trajectories of *attB* (grey, *n* = 59). (**B**) Time-averaged mean squared displacement in the transversal (left panel) and longitudinal (right panel) directions ($MS{D}_x\,$and$\ MS{D}_y$ respectively) of individual trajectories of *attB* (gray, *n* = 59). The effective diffusion coefficient $D\,$and exponent $\alpha$ defined by $MSD\ = \ D{t}^\alpha$ corresponding to the mean $MSD$ over all trajectories (black line) are given in each panel in black. (**C**) Same as in (B), in cells treated with DNP (*n* = 38).

As a first step to characterize the dynamics of *attB* in live cells, we calculated the mean square displacement ($MSD\ = \langle ({{\boldsymbol{r}}( {t + \tau } ) - {\boldsymbol{r}}(t} ){)}^2\rangle\,$) of 2D trajectories (Figure 5A), where ${\boldsymbol{r}}( t )$ is the position of the locus at time $t$, and $\tau$ is a delay time. A power law fit $MSD\ = \ D{\tau }^\alpha$ of the mean of the $MSD$ traces yields$\ \alpha \ = \ 0.36 \pm 0.02$$\ \alpha \ = \ 0.36 \pm 0.02$, indicating subdiffusive behavior (0<$ \alpha$<1). Here, $D$ is an apparent diffusion coefficient and $\alpha$ is a scaling exponent. The value of $\alpha \,$is comparable to those obtained at different chromosomal loci in *E. coli* (15,17,31). *B. subtilis* is a rod-shaped bacterium that grows along its longitudinal (*y*) axis and divides by binary fission. This structure has important functional implications for the organization of its chromosome and the segregation of copies into daughter cells (32,33). We inquired how this built-in asymmetry between length and width manifests itself in the motion of *attB* and ICE*Bs1*, prior to integration. To this end, we calculated separately the mean square displacement along the longitudinal ($\langle( {y( {t + \tau } ) - y(t} ){)}^2\rangle$) and transversal ($\langle( {x( {t + \tau } ) - x(t} ){)}^2\rangle$) directions ($MS{D}_y$ and $MS{D}_x$ respectively), from data taken at a ∼4 s intervals (*n* = 60, Figure 5B). Fitting these with a power law dependence, we obtained a ∼1.7-fold difference between ${\alpha }_y\,$(0.426 ± 0.022) and $\ {\alpha }_x$ (0.247 ± 0.005), i.e. asymmetric behavior. Bacterial growth as measured by the cell length for the duration and conditions of the experimental runs is negligible: the relative change in length is 0.06 ± 0.03% (mean ± SE, 100 cells). Boundary effects are also negligible in both directions. Indeed, single realizations of the mean square displacements (shown in gray in Figure 5) do not exhibit a transition to a plateau, as it would be expected for sub-diffusion in a bounded environment (34). We note that the sub-diffusive behavior of integrated ICE*Bs1* is similar to that of *attB* loci measured in the same cells (Supplementary Figure S3).

We determined the contributions of metabolic activity and thermal fluctuations to the motion of *attB* (15). We added 2,4-dinitrophenol (DNP) to cells, which depletes a cell of ATP and GTP and measured movement of the two loci (14). Addition of DNP caused a drastic reduction in the power-law exponents of the mean square displacements of *attB* in both directions, and a moderate reduction in the effective diffusion coefficients (Figure 5C), indicating that the motion of *attB* is primarily driven by metabolic activity, and not by thermal fluctuations. This finding is consistent with previous results showing that the thermal contribution to the dynamics of chromosomal loci and plasmids in *E. coli* and *C. crescentus* is small (14), and with slower vacuole motion in acanthamoebae as a result of myosin II inhibition (35).

In addition to global metabolic activity, the motion of chromosomal sites is driven on long timescales by genome replication and segregation. To determine the relative contribution of DNA replication and segregation to the subdiffusive behavior of *attB*, we inhibited DNA replication with 6-(*p*-hydroxyphenylazo)-uracil (HPUra), a specific inhibitor of DNA polymerase III (36). We show both $MS{D}_x$ and $MS{D}_y$ in Supplementary Figure S4A. We found that the values of the power law exponents and effective diffusion constants were comparable to those in untreated cells (Figure 5), indicating that the movements we observed of *attB* were not related to DNA replication.

**Figure 6.**
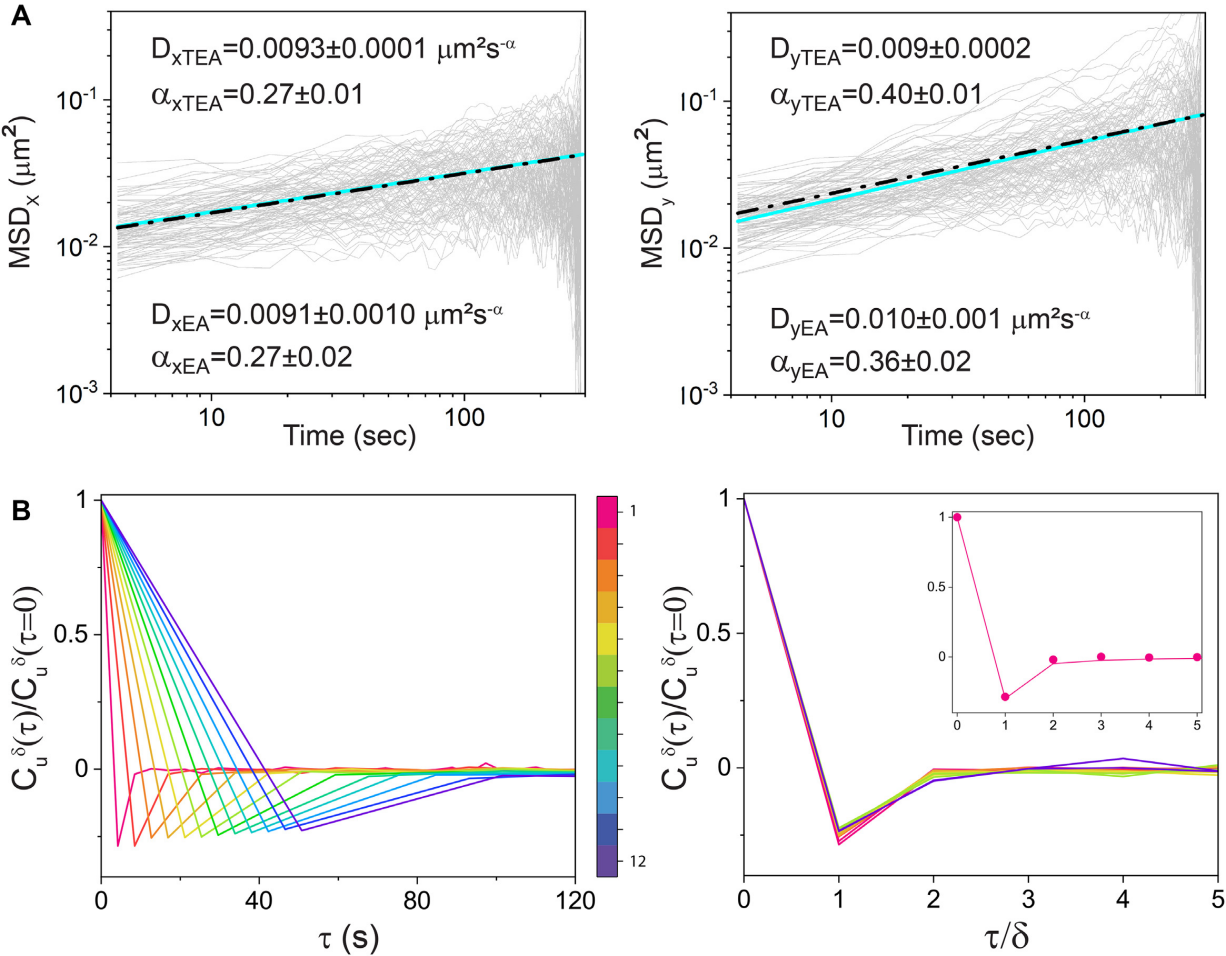
Dynamics of *attB* is ergodic. (**A**) Time-averaged (TEA) mean square displacements of *attB* (grey) in the longitudinal and transversal directions ($MS{D}_x$ and $MS{D}_y$ respectively), plotted as a function of time (*n* = 50). The straight line (cyan) is a power law fit to the mean $MSD$ of the respective panel. The dot-dashed straight line (black) is a power law fit to the mean of ensemble-averaged $MSD$ of *attB* in the longitudinal and transversal directions (EA). The respective values of the diffusion coefficients and power law exponents are given in each panel for both TAE and AE fits. (**B**) Left panel: normalized velocity autocorrelation function $C_u^\delta ( \tau )/C_u^\delta ( 0 )\,$of *attB* trajectories as a function of time, for different lag times $\ \delta$, which are multiples of the interval between successive frames (4.23 s). The velocity $\bar{u}\,$between positions separated by intervals $\delta \,$ranging from 4 to 40 s as indicated by the colour scheme bar. The colour scheme represents the values of $\delta$ from small (red) to large (purple). Right panel: same velocity autocorrelation function as in the corresponding left panel, plotted against rescaled time$\ \tau /\delta$. Inset: experimentally measured $C_u^1( \tau )/C_u^1( 0 )$ (circles) and Eq. (2) for$\ \alpha \ = \ 0.36$.

Three non-exclusive mechanisms are known to give rise to subdiffusive behavior: continuous time random walks (CTRW); obstructed diffusion (OD); and fractional Brownian motion (FLM) (37). In continuous time random walks, motion consists of a sequence of alternating jumping and binding events with a broad distribution of binding times (38–40). Obstructed diffusion reflects motion within a crowded environment such as the cell's interior (41). Fractional Brownian motion reflects a viscoelastic response stemming both from the cytoplasm, and the chromosome itself. As a first step to elucidate which of these mechanism(s) determines *attB* dynamics, we tested *attB* trajectories for ergodicity, comparing the time-averaged$\ MSD$ with the ensemble-averaged $MSD$ (Figure 6A). For an ergodic process, time averages are equivalent to ensemble averages, in particular for the $MSD$ (42). Both calculations yielded the same result within experimental error, supporting the notion that a continuous time random walk mechanism can be ruled out (43,44). Next, to discriminate between the obstructed diffusion and fractional Brownian motion mechanisms, we calculated the velocity auto-correlation function $C_u^\delta ( \tau )\,$(13,42,45):


(1) $$\begin{equation*}C_u^\delta \ \left( \tau \right) = \langle\bar{u}\left( {t + \tau } \right) \cdot \bar{u}\left( t \right) \rangle\end{equation*}$$

where $\ \bar{u}\ ( t ) = ( {\bar{r}( {t + \delta } ) - \bar{r}( t )} )\ /\delta$, is an velocity and $\delta$ is the time interval over which the velocity is calculated (a multiple of the mean interval between successive frames) and $\tau$ is a lag time. We show in Figure 6B the ensemble average of the time-averaged normalized $C_u^\delta ( \tau )$ for various values of$\,\delta \,$. For all temporal resolutions, the different $C_u^\delta ( \tau )$ display a negative peak at$\ \tau \ = \ \delta$, indicating that the elastic properties of the intracellular medium induce anti-persistent behavior, a characteristic of a viscoelastic environment. Since obstructed diffusion produces trajectories with uncorrelated steps, and therefore no antipersistent behavior, we conclude that fractional Brownian motion is the model that best describes the dynamical characteristics of *attB* motion. In fact, fractional Brownian motion describes quantitatively the behavior of the velocity autocorrelation function. Plotting the $C_u^\delta ( \tau )$ for different $\delta \,$as a function of the rescaled time lag $\ \xi \ = \ \tau /\delta$ results in a collapse of all velocity autocorrelation functions into a universal curve, indicating that the dynamics of the *attB* locus is self-similar (Figure 6B). This self-similar curve can be well fitted by the predicted $C_u^\delta ( \tau )\,$ from fractional Brownian motion (45,46):


(2) $$\begin{equation*}C_u^\delta \ \left( \xi \right) = \left\{ {{{\left( {\xi + 1} \right)}}^\alpha + {{\left| {\xi - 1} \right|}}^\alpha - 2{\xi }^\alpha } \right\} /2\end{equation*}$$

as shown in Figure 6B (inset). Depletion of ATP and GTP by DNP results in stronger viscoelastic behavior (Supplementary Figure S5A) than in the untreated case (Figure 6B), consistently with the observed lower values of the power law exponents $\alpha$ (Figure 5C).

### Tracking the motion of ICE*Bs1* in transconjugants

We also characterized the movement of ICE*Bs1* in transconjugants. Figure 3 displays representative ICE*Bs1* trajectories, color-coded according to time. The mean square displacements in transversal and longitudinal directions $MS{D}_x$ and $MS{D}_y\,$are shown in Supplementary Figure S6A. The dynamics are subdiffusive and the large asymmetry in the motion in transversal and longitudinal directions is reflected in a ∼4-fold difference in the exponents ${\alpha }_x$ and $\ {\alpha }_y$. Since the dynamical characterization of ICE*Bs1* was carried out in the same cells as that for *attB*, we assume their dynamics is also governed by the viscoelastic nature of the cytoplasm. The heterogeneity of ICE*Bs1* motion (Figure 3 and Supplementary Figure S2), and in particular the existence of both local and cell-scale explorations, indicates that ICE*Bs1* dynamics are heavily influenced by the chromosomal structure in the transconjugant.

### Effects of perturbing chromosomal structure on ICE*Bs1* dynamics

Further insights into the role of chromosomal structure can be gleaned by following the behavior of ICE*Bs1* and *attB* in cells treated with antibiotics that inhibit either transcription or translation, known to affect chromosomal structure (47–52). Specifically, we used rifampicin, an inhibitor of transcription that is known to induce nucleoid expansion (51,53–56), and chloramphenicol, an inhibitor of translation that is known to cause nucleoid compaction (53,56–58). Note that blocking transcription by treatment with rifampicin essentially also prevents translation after degradation of pre-existing mRNA.

Rifampicin significantly affected ICE*Bs1* dynamics. We found that in 76 ± 3% (mean ± SE, *n* = 213) of cells, ICE*Bs1* displayed highly mobile behavior spanning most of the cell (Supplementary Figure S6B), in contrast to the untreated case (14 ± 2%, *n* = 868). Similarly to the untreated case, this motion is visibly episodic, with local explorations interrupted by rapid longitudinal motion (Supplementary Figure S6B). This behavior gives rise to a very large asymmetry between the transversal and longitudinal directions. Furthermore, an $MSD$ analysis shows a ∼2–3-fold increase in the apparent diffusion coefficients in both directions (Supplementary Figure S6B), relative to untreated cells (Supplementary Figure S6A). However, no significant differences were obtained in the values of the power law exponents. Finally, it is worth noting that the larger mobility of ICE*Bs1* allowed it to carry out an increased exploration of regions where *attB* loci were more likely to be found (Figure 4B).

In contrast to the effects on ICE*Bs1*, the effects on the dynamics of *attB* loci are to increase the effective diffusion constants in both longitudinal and transversal directions (Supplementary Figure S4B). An analysis of the dynamics of *attB* loci using the velocity auto-correlation function $C_u^\delta ( \tau )$ shows that rifampicin treatment preserves the essential features observed in untreated cells (Supplementary Figure S5C).

In contrast to rifampicin, chloramphenicol induced a marked reduction in the range of motion of ICE*Bs1* (Supplementary Figure S6C). In fact, the extent of the motion was similar to that of *attB*. The percentage of cells in which ICE*Bs1* exhibited motion without being integrated, was 23 ± 1%, about 2-fold less than that in untreated cells (Supplementary Figure S6A). The effects of chloramphenicol on *attB* were also significant (Supplementary Figure S4C), abolishing the asymmetry in longitudinal and transversal directions observed in the untreated case: a mean square displacement analysis of *attB* trajectories showed that within experimental error, ${\alpha }_y = \ 0.30 \pm 0.01$ and $\ \ {\alpha }_x = \ 0.309 \pm 0.006$ (Supplementary Figure S6C). This result is consistent with the known compaction of the chromosome by chloramphenicol, which was shown previously to stabilize mRNAs and induce accumulation of rRNAs (56). We tested whether chromosome compaction would also enable larger explorations of the cell's interior by ICE*Bs1*. This notion is borne out by the results shown in Figure 4C. Furthermore, the likelihood of finding *attB* near a cell center was observed to be higher than in the untreated case, in line with the reported compaction of the chromosome (53,56–58). None of the antibiotic treatments nor HPUra, affected the behavior of the velocity auto-correlation functions of *attB* (Supplementary Figure S5B–D). Based on the different results with rifampicin and chloramphenicol, we conclude that the effects of blocking transcription with rifampicin were not due to the pleiotropic effects of blocking translation. Together, our results indicate that perturbations of chromosome structure and alteration of the crowded nature of the cytoplasm by rifampicin and chloramphenicol affect the dynamics of ICE*Bs1*, as it searches for its integration site.

## DISCUSSION

A striking feature that emerges from the experiments reported in this work is the stark contrast between the large variability of dynamical behaviors that ICE*Bs1* can display as it searches the *B. subtilis* chromosome, and the quasi-regular motion of the chromosomal site of integration (*attB*). As discussed below, we believe that the structure of the bacterial chromosome (59) likely dictates the dynamical characteristics of ICE*Bs1* movement.

Recent experiments and models of chromosomal configurations and segregation in *B. subtilis* have revealed that on large scales, the chromosome is arranged longitudinally, with bristle-like plectonemic loops topologically isolated at their base, protruding in the transversal (radial) direction, in a bottle-brush configuration (29,32,47,59–61). The pronounced asymmetry in the $MSD$ of *attB* loci we observed, with larger displacements along the longitudinal than in the transversal direction is consistent with this structure: while bristles may easily fluctuate together along the longitudinal direction, displacements in the transversal radial direction are more constrained by the embedding of the *attB* site within the bottle-brush structure. This asymmetric behavior, observed at subcellular scales, may be more general and contrasts with asymmetric mean square displacements detected at extracellular scales during cellular motion in gradients (62). The mean square displacement power law exponents of *attB* trajectories are indicative of sub-diffusive behavior, and are consistent with previous observations on the motion of chromosomal loci in other bacteria such as *E. coli* and *C. crescentus* (17,63).

Furthermore, our analysis indicates that the dynamics of *attB* are ergodic and that sub-diffusivity is due primarily to the viscoelastic nature of the nucleoid (64). *attB* constitutes a moving target driven by active processes (29,63,65) and the spatial extent of its motion is limited to well-defined subcellular regions centered around 1/4 and 3/4 of the cell's length. The positional distribution of ICE*Bs1* that has not yet integrated along the longitudinal coordinate display overlap with *attB* positions that can allow ICE*Bs1* to find its target for integration. Simulations have provided evidence that sub-diffusion massively enhances the probability of finding a target despite being slower than normal diffusion, making a search process more reliable (66,67).

In contrast to the relatively regular dynamical behavior of *attB*, ICE*Bs1* motion was characterized by significant dynamical heterogeneity, ranging from rapid large-scale motion that spanned a large portion of the cell length, to localized motion, e.g. near cell centers, to quiescent behavior near cell poles. These behaviors are in addition to that of the integrated element, which behaves similarly to *attB*. The sub-diffusive motion of ICE*Bs1* is driven primarily by out-of-equilibrium, athermal, metabolic fluctuations of the cytoplasm (14), and by transient integrase-mediated associations between the recipient chromosome and ICE*Bs1* (68).

It is difficult to reconcile the episodic rapid, large-scale motion of ICE*Bs1* as it criss-crosses a cell with penetration and reptation through brush bristles. We hypothesize that during such episodes, ICE*Bs1* localizes primarily to nucleoid fringes where its motion is relatively unhindered. This picture is consistent with entropic eviction of plasmids from the nucleoid, observed in experiments in *E. coli* and simulations (69). The observed localization and motion of ICE*Bs1*s near cell centers between sister copies of *attB* indicates that brush density there is reduced, as expected between the two nucleoids of incipient daughter cells, and is indicative of an entropic price for ICE*Bs1* to penetrate into the respective bottlebrush structures.

The importance of chromosomal structure in determining ICE*Bs1* dynamics in a transconjugant is further substantiated by the effects of perturbations to chromosomal structure. Inhibition of transcription by rifampicin is known to decompact the chromosome, which has been observed to span all the cytoplasm up to the membrane (51,53). The effects of chromosomal structure decompaction are clearly observed in the dynamical behavior of ICE*Bs1*. Following treatment with rifampicin, the proportion of cells in which ICE*Bs1* displays fast, free motion is significantly larger than in the untreated case (∼5-fold), as measured by the apparent diffusion coefficient. These features are consistent with ICE*Bs1* motion within an open, porous chromosomal mesh. The mesh size $\xi \,$of the bacterial nucleoid regarded as a polymer network has been measured recently to be $\xi\sim$50 nm in the case of *E. coli* (56). Assuming that the ∼20 kb DNA of ICE*Bs1* undergoes similar compaction as the bacterial genome, and that $\xi \,$in *B. subtilis* is similar to that of *E. coli*, one can estimate the size of ICE*Bs1* within the cytoplasm. The estimate yields 50–70 nm, a size that is of the same order of magnitude as $\xi$, enabling the search for *attB* across the cell. The larger percentage of cells displaying large scale high mobility dynamics of ICE*Bs1* with rifampicin is in line with these notions. One may speculate that larger ICEs will display a lower efficiency of search and integration than ICE*Bs1*. We also note that in eukaryotic cells, objects whose size is smaller than 50 nm -below the pore size of the cytoplasmic network- behave like Brownian particles diffusing in a medium of low viscosity (70). Effects of rifampicin on the changes in chromosomal structure are also noticeable in the dynamical behavior of *attB*. The comparable values of ${\alpha }_x\,$and ${\alpha }_y$ indicate a partial loss of the anisotropy of motion observed in the untreated case. Nonetheless, residual anisotropy still remains in the apparent diffusion coefficients (${D}_y >{D}_x$). Note that the values of these coefficients are about three-fold larger than in the untreated case, indicating larger local mobility of chromosomal segments. These results are consistent with previous evidence showing that rifampicin effects reduces the static fraction of SMC proteins in *B. subtilis* and increases its diffusion (71).

Whereas the compaction of the chromosome by inhibition of translation, and the consequent availability of a larger nucleoid-free volume might have led to greater mobility of ICE*Bs1*, the results of our experiments indicate that this is not the case. The drastic reduction in the proportion of highly mobile ICE*Bs1*s observed after addition of chloramphenicol is instead consistent with evidence from a recent study in which it was shown that nucleoid compaction by translation inhibition is caused by an increase in RNA levels(56), since chloramphenicol results in stabilization of mRNAs (72–74), and in higher levels of free rRNAs (75,76). The higher levels of RNA in nucleoid-free regions, crowding the intracellular medium may also hinder significantly ICE*Bs1*s mobility. In addition, a compacted chromosome may also severely reduce accessibility and search for *attB* due to a decrease in mesh size.

Based on the observed response of ICE*Bs1* to changes in the compaction state of the bacterial chromosome induced by antibiotics, we anticipate that the dynamics of the search process might also be affected by the size of the ICE itself. ICE*Bs1* is ∼21 kb, but ICEs range from ∼12 kb to ∼500 kb (77,78). ICEs are increasingly used for genetic engineering and analyses, particularly in undomesticated bacteria (79). Understanding the effects of ICE size on the search process will be important in improving their use and in understanding the physical constraints on target site selection.

The search mechanism used by ICE*Bs1* differs substantially from that used by lambda phage to find its attachment site during lysogenization (80). In sharp contrast to ICE*Bs1*s, which can span all a recipient's interior until the integration site *attB* is found, phage DNA remains confined near its point of entry primarily near the poles in an infected cell. There, the integration site approaches it, driven by the replication and segregation of the host genome (80). Thus, borrowing the language of the rendezvous problem of operations research, phage lambda employs a ‘Wait for Mummy’ strategy (WFM) (81), in order to find its integration site. We hypothesize that ICE*Bs1* motion may be required to enable encounter with its integration site, as ICE*Bs1* can likely enter a recipient cell at any point along the cell's contour (16), in contrast to lambda DNA that enters preferentially at the cell poles. The WFM strategy is rarely optimal (82), and yet lysogenization of lambda phage can reach a very high efficiency (∼60%).

While the variegated dynamical behavior of ICE*Bs1* differs considerably from that of lambda phage, our experiments following the search at the single-cell level reveal that the efficiency of target location by ICE*Bs1* for an integration site is very high. Indeed, the fractal dimension associated with the trajectory of an ICE*Bs1* prior to integration is ${d}_w = 2/\alpha \,$, and since $\alpha$ values are smaller than 1, ${d}_w >2$, i.e. larger than the embedding space. This guarantees that the path explored by the ICE*Bs1* is space-filling, a crucial feature to ensure a thorough search for the *attB* target. In the unlikely case in which an integration site is not found, the rapid cell to cell spreading of ICE*Bs1* along chains, common in microbial communities on surfaces, increases further the efficiency of conjugation, amplifying the number of cells that acquire conjugative mobile genetic elements (16,83,84).

The task faced by an ICE as it searches for a unique site of integration along a host chromosome is highly challenging. Bacterial chromosomes are typically millions of base pairs long, and the number of possible addresses is thus very large. Furthermore, the search is compounded by the complex nature of the crowded, viscoelastic bacterial interior and its non-equilibrium, active nature. Compared to transcription factors searching for cognate binding sites, the small number of ICEs in a recipient is small and their size is about an order of magnitude larger. Yet, the search is efficient and target sites are located within physiologically relevant timescales. How is this task accomplished?

Our results provide key insights about the strategy used by integrating conjugating DNA elements in their search. First, far from being a diffusive process, the search is episodic, allowing for compact local explorations interspersed by periods in which ICE motion is fast, crisscrossing the cell within seconds-long intervals. This highly heterogeneous dynamic behavior and the attendant search strategy, is consistent with bacterial chromosome structure, and underscores the fact that heterogeneity is essential in bacterial subcellular organization, despite the fact that a bacterium consists of only one compartment. Second, the motion of both the integration site and ICE is subdiffusive and remarkably, highly anisotropic. Our observations indicate that the anisotropy reflects bacterial chromosome structure and not simply the stadium-like, elongated shape of a cell and its boundaries. From a theoretical perspective, our work stimulates the quest for an anomalous diffusion model that can characterize the anisotropy in the diffusion exponents. The interest extends beyond the specific context of ICEs and *attBs* motion, to active systems in which the anisotropy might prove crucial for uncovering details that are lost in the steady-state (85,86).

Our results raise many fundamental evolutionary questions. For example, has the size of ICE*Bs1*, or other long DNA molecules imported into a cell during horizontal gene transfer processes been tuned by evolutionary pressures? Answering this question is critical to optimize the use of ICEs for the genetic modification of undomesticated bacteria (79). Has the site of integration along the chromosome been optimized? In this context it is important to stress that the degree of compaction varies locally along chromosomal coordinates, affecting the size mesh, the search by ICE*Bs1* and its access to the integration locus. To what extent is anisotropic dynamical behavior advantageous to search process? Future studies could address these questions and the extent to which other aspects of the search were honed by evolution.

## DATA AVAILABILITY

The software for segmentation is available at https://github.com/ferinat/Bacillus-Analysis-Public.

## Supplementary Material

gkad068_Supplemental_Files

## References

[1] Wiedenbeck J. , Cohan F.M. Origins of bacterial diversity through horizontal genetic transfer and adaptation to new ecological niches. FEMS Microbiol. Rev. 2011; 35:957–976. 21711367 10.1111/j.1574-6976.2011.00292.x

[2] Guglielmini J. , Quintais L., Garcillán-Barcia M.P., de la Cruz F., Rocha E.P.C. The repertoire of ICE in prokaryotes underscores the unity, diversity, and ubiquity of conjugation. PLoS Genet. 2011; 7:e1002222. 21876676 10.1371/journal.pgen.1002222 PMC3158045

[3] Touchon M. , Rocha E.P.C. Coevolution of the organization and structure of prokaryotic genomes. Cold Spring Harb. Perspect. Biol. 2016; 8:a018168. 26729648 10.1101/cshperspect.a018168 PMC4691797

[4] Carraro N. , Burrus V. Biology of three ICE families: SXT/R391, ICE*Bs1*, and ICE*St1*/ICE*St3*. In *Mobile DNA III*. Am. Soc. Microbiol. 2014; 2:289–309.

[5] Auchtung J.M. , Aleksanyan N., Bulku A., Berkmen M.B. Biology of ICEBs1, an integrative and conjugative element in *Bacillus subtilis*. Plasmid. 2016; 86:14–25. 27381852 10.1016/j.plasmid.2016.07.001

[6] Menard K.L. , Grossman A.D. Selective pressures to maintain attachment site specificity of integrative and conjugative elements. PLoS Genet. 2013; 9:e1003623. 23874222 10.1371/journal.pgen.1003623 PMC3715440

[7] Halford S.E. , Marko J.F. How do site-specific DNA-binding proteins find their targets?. Nucleic Acids Res. 2004; 32:3040–3052. 15178741 10.1093/nar/gkh624 PMC434431

[8] Dey P. , Bhattacherjee A. Role of macromolecular crowding on the intracellular diffusion of DNA binding proteins. Sci Rep. 2017; 8:844. 10.1038/s41598-017-18933-3 PMC5770392 29339733

[9] Barzel A. , Kupiec M. Finding a match: how do homologous sequences get together for recombination?. Nat. Rev. Genet. 2008; 9:27–37. 18040271 10.1038/nrg2224

[10] Tal A. , Arbel-Goren R., Costantino N., Court D.L., Stavans J. Location of the unique integration site on an *Escherichia coli* chromosome by bacteriophage lambda DNA in vivo. Proc. Natl. Acad. Sci. U.S.A. 2014; 111:7308–7312. 24799672 10.1073/pnas.1324066111 PMC4034188

[11] Jones D.L. , Leroy P., Unoson C., Fange D., Ćurić V., Lawson M.J., Elf J. Kinetics of dCas9 target search in *Escherichia coli*. Science. 2017; 357:1420–1424. 28963258 10.1126/science.aah7084 PMC6150439

[12] Vink J. , Martens K., Vlot M., McKenzie R., Almendros C., Bonilla B., Brocken D., Hohlbein J., Brouns S. Direct visualization of native CRISPR target search in live bacteria reveals Cascade DNA surveillance mechanism. Mol. Cell. 2020; 77:39–50. 31735642 10.1016/j.molcel.2019.10.021

[13] S Mogre S. , Brown A.I., Koslover E.F. Getting around the cell: physical transport in the intracellular world. Phys. Biol. 2020; 17:061003. 32663814 10.1088/1478-3975/aba5e5

[14] Parry B.R. , Surovtsev I.V., Cabeen M.T., O’Hern C.S., Dufresne E.R., Jacobs-Wagner C. The bacterial cytoplasm has glass-like properties and is fluidized by metabolic activity. Cell. 2014; 156:183–194. 24361104 10.1016/j.cell.2013.11.028 PMC3956598

[15] Weber S.C. , Spakowitz A.J., Theriot J.A. Nonthermal ATP-dependent fluctuations contribute to the in vivo motion of chromosomal loci. Proc. Natl. Acad. Sci. U.S.A. 2012; 109:7338–7343. 22517744 10.1073/pnas.1119505109 PMC3358901

[16] Babic A. , Berkmen M.B., Lee C.A., Grossman A.D. Efficient gene transfer in bacterial cell chains. MBio. 2011; 2:e00027-11. 21406598 10.1128/mBio.00027-11 PMC3055163

[17] Weber S.C. , Spakowitz A.J., Theriot J.A. Bacterial chromosomal loci move subdiffusively through a viscoelastic cytoplasm. Phys. Rev. Lett. 2010; 104:238102. 20867274 10.1103/PhysRevLett.104.238102 PMC4929007

[18] Espeli O. , Mercier R., Boccard F. DNA dynamics vary according to macrodomain topography in the *E. coli* chromosome. Mol. Microbiol. 2008; 68:1418–1427. 18410497 10.1111/j.1365-2958.2008.06239.x

[19] Fiebig A. , Keren K., Theriot J.A. Fine-scale time-lapse analysis of the biphasic, dynamic behaviour of the two *Vibrio cholerae* chromosomes. Mol. Microbiol. 2006; 60:1164–1178. 16689793 10.1111/j.1365-2958.2006.05175.x PMC2779472

[20] Perego M. , Spiegelman G.B., Hoch J.A. Structure of the gene for the transition state regulator, abrB: regulator synthesis is controlled by the *spo0A* sporulation gene in *Bacillus subtilis*. Mol. Microbiol. 1988; 2:689–699. 3145384 10.1111/j.1365-2958.1988.tb00079.x

[21] Smith J.L. , Goldberg J.M., Grossman A.D. Complete genome sequences of *Bacillus subtilis* subsp. *subtilis* laboratory strains JH642 (AG174) and AG1839. Genome Announc. 2014; 2:663–677. 10.1128/genomeA.00663-14 PMC4082004 24994804

[22] Auchtung J.M. , Lee C.A., Monson R.E., Lehman A.P., Grossman A.D. Regulation of a *Bacillus subtilis* mobile genetic element by intercellular signaling and the global DNA damage response. Proc. Nat. Acad. Sci. U.S.A. 2005; 102:12554–12559. 10.1073/pnas.0505835102 PMC1194945 16105942

[23] Lee P.S. , Grossman A.D. The chromosome partitioning proteins Soj (ParA) and Spo0J (ParB) contribute to accurate chromosome partitioning, separation of replicated sister origins, and regulation of replication initiation in *Bacillus subtilis*. Mol. Microbiol. 2006; 60:853–869. 16677298 10.1111/j.1365-2958.2006.05140.x

[24] Shaner N. , Lin M., McKeown M., Steinbach P., Hazelwood K., Davidson M., Tsien R. Improving the photostability of bright monomeric orange and red fluorescent proteins. Nat. Methods. 2008; 5:545–551. 18454154 10.1038/nmeth.1209 PMC2853173

[25] Lau I.F. , Filipe S.R., Søballe B., Økstad O.A., Barre F.X., Sherratt D.J. Spatial and temporal organization of replicating *Escherichia coli* chromosomes. Mol. Microbiol. 2003; 49:731–743. 12864855 10.1046/j.1365-2958.2003.03640.x

[26] Harwood C. , Cutting S. Molecular Biological Methods for *Bacillus*. Wiley. 1990; NY Chichester.

[27] Gibson D.G. , Young L., Chuang R.-Y., Craig Venter J., Hutchison C.A. III, Smith H.O Enzymatic assembly of DNA molecules up to several hundred kilobases. Nat. Methods. 2009; 6:343–345. 19363495 10.1038/nmeth.1318

[28] Golding I. , Cox E.C. Physical nature of bacterial cytoplasm. Phys. Rev. Lett. 2006; 96:098102. 16606319 10.1103/PhysRevLett.96.098102

[29] Wang X. , Llopis P.M., Rudner D.Z. Bacillus subtilis chromosome organization oscillates between two distinct patterns. Proc. Natl. Acad. Sci. U.S.A. 2014; 111:12877–12882. 25071173 10.1073/pnas.1407461111 PMC4156703

[30] Kuhlman T.E. , Cox E.C. Gene location and DNA density determine transcription factor distributions in *Escherichia coli*. Mol. Syst. Biol. 2012; 8:610. 22968444 10.1038/msb.2012.42 PMC3472691

[31] Javer A. , Long Z., Nugent E., Grisi M., Siriwatwetchakul K., Dorfman K.D., Cicuta P., Lagomarsino M.C. Short-time movement of *E. coli* chromosomal loci depends on coordinate and subcellular localization. Nat. Commun. 2013; 4:3003. 23764719 10.1038/ncomms3003

[32] Marbouty M. , Le Gall A., Cattoni D.I., Cournac A., Koh A., Fiche J.-B., Mozziconacci J., Murray H., Koszul R., Nollmann M. Condensin-and replication-mediated bacterial chromosome folding and origin condensation revealed by Hi-C and super-resolution imaging. Mol. Cell. 2015; 59:588–602. 26295962 10.1016/j.molcel.2015.07.020

[33] Wang X. , Le T.B.K., Lajoie B.R., Dekker J., Laub M.T., Rudner D.Z. Condensin promotes the juxtaposition of DNA flanking its loading site in *Bacillus subtilis*. Genes Dev. 2015; 29:1661–1675. 26253537 10.1101/gad.265876.115 PMC4536313

[34] Burov S. , Metzler R., Barkai E. Aging and nonergodicity beyond the Khinchin theorem. Proc. Nat. Acad. Sci. U.S.A. 2010; 107:13228–13233. 10.1073/pnas.1003693107 PMC2922158 20624984

[35] Reverey J.F. , Jeon J.H., Bao H., Leippe M., Metzler R., Selhuber-Unkel C. Superdiffusion dominates intracellular particle motion in the supercrowded cytoplasm of pathogenic *Acanthamoeba castellanii*. Sci. Rep. 2015; 51:11690. 10.1038/srep11690 PMC5155589 26123798

[36] Brown N. 6-(p-Hydroxyphenylazo)-uracil: a selective inhibitor of host DNA replication in phage-infected *Bacillus subtilis*. Proc. Nat. Acad. Sci. U.S.A. 1970; 67:1454–1461. 10.1073/pnas.67.3.1454 PMC283373 4992015

[37] Lutz E. Fractional Langevin equation. Fractional Dynamics: Recent Advances. 2011; World Scientific Publishing Co 285–305.

[38] Montroll E. , Weiss G. Random walks on lattices. II. J. Math. Phys. 1965; 6:167–181.

[39] Saxton M.J. Anomalous diffusion due to binding: a Monte Carlo study. Biophys. J. 1996; 70:1250–1262. 8785281 10.1016/S0006-3495(96)79682-0 PMC1225051

[40] Scher H. , Montroll E.W. Anomalous transit-time dispersion in amorphous solids. Phys. Rev. B. 1975; 12:2455–2477.

[41] Saxton M.J. Anomalous diffusion due to obstacles: a Monte Carlo study. Biophys. J. 1994; 66:394–401. 8161693 10.1016/s0006-3495(94)80789-1 PMC1275707

[42] Zhang Y. , Dudko O.K. First-passage processes in the genome. Annu. Rev. Biophys. 2016; 45:117–134. 27391924 10.1146/annurev-biophys-062215-010925

[43] He Y. , Burov S., Metzler R., Barkai E. Random time-scale invariant diffusion and transport coefficients. Phys. Rev. Lett. 2008; 101:058101. 18764430 10.1103/PhysRevLett.101.058101

[44] Deng W. , Barkai E. Ergodic properties of fractional Brownian-Langevin motion. Phys. Rev. E - Stat. Nonlinear, Soft Matter Phys. 2009; 79:011112. 10.1103/PhysRevE.79.011112 19257006

[45] Burov S. , Jeon J.-H., Metzler R., Barkai E. Single particle tracking in systems showing anomalous diffusion: the role of weak ergodicity breaking. Phys. Chem. Chem. Phys. 2011; 13:1800–1812. 21203639 10.1039/c0cp01879a

[46] Backlund M. , Joyner R., Moerner W. Chromosomal locus tracking with proper accounting of static and dynamic errors. Phys. Rev. E. 2015; 91:062716. 10.1103/PhysRevE.91.062716 PMC4533921 26172745

[47] Wang X. , Brandão H.B., Le T.B.K., Laub M.T., Rudner D.Z. Bacillus subtilis SMC complexes juxtapose chromosome arms as they travel from origin to terminus. Science. 2017; 355:524–527. 28154080 10.1126/science.aai8982 PMC5484144

[48] Stracy M. , Lesterlin C., De Leon F., Uphoff S., Zawadzki P., Kapanidis A. Live-cell superresolution microscopy reveals the organization of RNA polymerase in the bacterial nucleoid. Proc. Natl. Acad. Sci. U.S.A. 2015; 112:E4390–E4399. 26224838 10.1073/pnas.1507592112 PMC4538611

[49] Wlodarski M. , Mancini L., Raciti B., Sclavi B., Lagomarsino M.C., Cicuta P. Cytosolic crowding drives the dynamics of both genome and cytosol in *Escherichia coli* challenged with sub-lethal antibiotic treatments. Iscience. 2020; 23:101560. 33083729 10.1016/j.isci.2020.101560 PMC7522891

[50] Wlodarski M. , Raciti B., Kotar J., Cosentino Lagomarsino M., Fraser G.M., Cicuta P. Both genome and cytosol dynamics change in *E. coli* challenged with sublethal rifampicin. Phys. Biol. 2017; 14:015005. 28207419 10.1088/1478-3975/aa5b71

[51] Cabrera J.E. , Jin D.J. The distribution of RNA polymerase in *Escherichia coli* is dynamic and sensitive to environmental cues. Mol. Microbiol. 2003; 50:1493–1505. 14651633 10.1046/j.1365-2958.2003.03805.x

[52] Bylund J.E. , Haines M.A., Piggot P.J., Higgins M.L. Axial filament formation in *Bacillus subtilis*: induction of nucleoids of increasing length after addition of chloramphenicol to exponential-phase cultures approaching stationary phase. J. Bacteriol. 1993; 175:1886–1890. 7681431 10.1128/jb.175.7.1886-1890.1993 PMC204252

[53] Bakshi S. , Siryaporn A., Goulian M., Weisshaar J.C. Superresolution imaging of ribosomes and RNA polymerase in live *Escherichia coli* cells. Mol. Microbiol. 2012; 85:21–38. 22624875 10.1111/j.1365-2958.2012.08081.x PMC3383343

[54] Yang D. , Männik J., Retterer S.T., Männik J. The effects of polydisperse crowders on the compaction of the *Escherichia coli* nucleoid. Mol. Microbiol. 2020; 113:1022–1037. 31961016 10.1111/mmi.14467 PMC7237313

[55] Sun Q. , Margolin W. Effects of perturbing nucleoid structure on nucleoid occlusion-mediated toporegulation of FtsZ ring assembly. J. Bacteriol. 2004; 186:3951–3959. 15175309 10.1128/JB.186.12.3951-3959.2004 PMC419936

[56] Xiang Y. , Surovtsev I., Chang Y., Govers S., Parry R., Liu J., Jacobs-Wagner C. Interconnecting solvent quality, transcription, and chromosome folding in *Escherichia coli*. Cell. 2021; 184:3626–3642. 34186018 10.1016/j.cell.2021.05.037

[57] Cabrera J.E. , Cagliero C., Quan S., Squires C.L., Jin D.J. Active transcription of rRNA operons condenses the nucleoid in *Escherichia coli*: examining the effect of transcription on nucleoid structure in the absence of transertion. J. Bacteriol. 2009; 191:4180–4185. 19395497 10.1128/JB.01707-08 PMC2698504

[58] Zimmerman S.B. Toroidal nucleoids in *Escherichia coli* exposed to chloramphenicol. J. Struct. Biol. 2002; 138:199–206. 12217658 10.1016/s1047-8477(02)00036-9

[59] Badrinarayanan A. , Le T.B.K., Laub M.T. Bacterial chromosome organization and segregation. Annu. Rev. Cell Dev. Biol. 2015; 31:171–199. 26566111 10.1146/annurev-cellbio-100814-125211 PMC4706359

[60] Zraly C.B. , Dingwall A.K. The chromatin remodeling and mRNA splicing functions of the Brahma (SWI/SNF) complex are mediated by the SNR1/SNF5 regulatory subunit. Nucleic Acids Res. 2012; 40:5975–5987. 22467207 10.1093/nar/gks288 PMC3401471

[61] Banigan E.J. , Mirny L.A. Loop extrusion: theory meets single-molecule experiments. Curr. Opin. Cell Biol. 2020; 64:124–138. 32534241 10.1016/j.ceb.2020.04.011

[62] Dieterich P. , Lindemann O., Moskopp M.L., Tauzin S., Huttenlocher A., Klages R., Chechkin A., Schwab A. Anomalous diffusion and asymmetric tempering memory in neutrophil chemotaxis. PLoS Comput. Biol. 2022; 18:e1010089. 35584137 10.1371/journal.pcbi.1010089 PMC9154114

[63] Campos M. , Jacobs-Wagner C. Cellular organization of the transfer of genetic information. Curr. Opin. Microbiol. 2013; 16:171–176. 23395479 10.1016/j.mib.2013.01.007 PMC3646911

[64] Kleckner N. , Fisher J.K., Stouf M., White M.A., Bates D., Witz G. The bacterial nucleoid: nature, dynamics and sister segregation. Curr. Opin. Microbiol. 2014; 22:127–137. 25460806 10.1016/j.mib.2014.10.001 PMC4359759

[65] El Najjar N. , Geisel D., Schmidt F., Dersch S., Mayer B., Hartmann R., Eckhardt B., Lenz P., Graumann P.L. Chromosome segregation in *Bacillus subtilis* follows an overall pattern of linear movement and is highly robust against cell cycle perturbations. Msphere. 2020; 5:e00255-20. 32554717 10.1128/mSphere.00255-20 PMC7300352

[66] Guigas G. , Weiss M. Sampling the cell with anomalous diffusion—the discovery of slowness. Biophys. J. 2008; 94:90–94. 17827216 10.1529/biophysj.107.117044 PMC2134854

[67] Weiss M. Crowding, diffusion, and biochemical reactions. Int. Rev. Cell Mol. Biol. 2014; 307:383–417. 24380600 10.1016/B978-0-12-800046-5.00011-4

[68] Johnson C.M. , Grossman A.D. Integrative and conjugative elements (ICEs): what they do and how they work. Annu. Rev. Genet. 2015; 49:577–601. 26473380 10.1146/annurev-genet-112414-055018 PMC5180612

[69] Planchenault C. , Pons M.C., Schiavon C., Siguier P., Rech J., Guynet C., Dauverd-Girault J., Cury J., Rocha E.P.C., Junier I. et al. Intracellular positioning systems limit the entropic eviction of secondary replicons toward the nucleoid edges in bacterial cells. J. Mol. Biol. 2020; 432:745–761. 31931015 10.1016/j.jmb.2019.11.027

[70] Etoc F. , Balloul E., Vicario C., Normanno D., Liße D., Sittner A., Piehler J., Dahan M., Coppey M. Non-specific interactions govern cytosolic diffusion of nanosized objects in mammalian cells. Nat. Mater. 2018; 17:740–746. 29967464 10.1038/s41563-018-0120-7

[71] Schibany S. , Hinrichs R., Hernández-Tamayo R., Graumann P.L. The major chromosome condensation factors Smc, HBsu, and Gyrase in *Bacillus subtilis* operate via strikingly different patterns of motion. Am. Soc. Microbiol. 2020; 5:e00817-20. 10.1128/mSphere.00817-20 PMC7485690 32907955

[72] Lopez P. , Marchand I., Yarchuk O., Dreyfus M. Translation inhibitors stabilize *Escherichia coli* mRNAs independently of ribosome protection. Proc. Nat. Acad. Sci. U.S.A. 1998; 95:6067–6072. 10.1073/pnas.95.11.6067 PMC27586 9600918

[73] Pato M.L. , Bennett P.M., Von Meyenburg K. Messenger ribonucleic acid synthesis and degradation in *Escherichia coli* during inhibition of translation. J. Bacteriol. 1973; 116:710–718. 4583248 10.1128/jb.116.2.710-718.1973 PMC285436

[74] Schneider E. , Blundell M., Kennell D. Translation and mRNA decay. MGG Mol. Gen. Genet. 1978; 160:121–129. 349350 10.1007/BF00267473

[75] Cole J.R. , Olsson C.L., Hershey J.W.B., Grunberg-Manago M., Nomura M. Feedback regulation of rRNA synthesis in *Escherichia coli*: requirement for initiation factor IF2. J. Mol. Biol. 1987; 198:383–392. 2448483 10.1016/0022-2836(87)90288-9

[76] Kurland C.G. , Maaløe O. Regulation of ribosomal and transfer RNA synthesis. J. Mol. Biol. 1962; 4:193–210. 14460744 10.1016/s0022-2836(62)80051-5

[77] Cury J. , Touchon M., Rocha E.P.C. Integrative and conjugative elements and their hosts: composition, distribution and organization. Nucleic Acids Res. 2017; 45:8943. 28911112 10.1093/nar/gkx607 PMC5587801

[78] Wozniak R. , Waldor M. Integrative and conjugative elements: mosaic mobile genetic elements enabling dynamic lateral gene flow. Nat. Rev. Microbiol. 2010; 8:552–563. 20601965 10.1038/nrmicro2382

[79] Brophy J. , Triassi A., Adams B., Renberg R., Stratis-Cullum D., Grossman A., Voigt C. Engineered integrative and conjugative elements for efficient and inducible DNA transfer to undomesticated bacteria. Nat. Microbiol. 2018; 3:1043–1053. 30127494 10.1038/s41564-018-0216-5

[80] Tal A. , Arbel-Goren R., Costantino N., Court D.L., Stavans J. Location of the unique integration site on an *Escherichia coli* chromosome by bacteriophage lambda DNA in vivo. Proc. Natl. Acad. Sci. U.S.A. 2014; 111:7308–7312. 24799672 10.1073/pnas.1324066111 PMC4034188

[81] Alpern S. Rendezvous search: a personal perspective. Oper. Res. 2002; 50:772–795.

[82] Alpern S. , Gal S. Introduction to rendezvous search. The Theory of Search Games and Rendezvous. 2003; Boston Kluwer Academic Publishers 165–172.

[83] Lécuyer F. , Bourassa J.-S., Gélinas M., Charron-Lamoureux V., Burrus V., Beauregard P.B. Biofilm formation drives transfer of the conjugative element ICE*Bs1* in *Bacillus subtilis*. Msphere. 2018; 3:e00473-18. 30258041 10.1128/mSphere.00473-18 PMC6158512

[84] Jones J.M. , Grinberg I., Eldar A., Grossman A.D. A mobile genetic element increases bacterial host fitness by manipulating development. Elife. 2021; 10:e65924. 33655883 10.7554/eLife.65924 PMC8032392

[85] Teza G. , Stella A. Exact coarse graining preserves entropy production out of equilibrium. Phys. Rev. Lett. 2020; 125:110601. 32975992 10.1103/PhysRevLett.125.110601

[86] Teza G. Out of Equilibrium Dynamics: From an Entropy of the Growth to the Growth of Entropy Production. 2020; University of Padova PhD thesis.

