# Supplementary material for: Target search by an imported conjugative DNA element for a unique integration site along a bacterial chromosome during horizontal gene transfer
Source: Nucleic Acids Res. 2023 Feb 10;51(7):3116–29. doi: 10.1093/nar/gkad068 (PMC10123120; doi:10.1093/nar/gkad068)
Supplement: gkad068_Supplemental_Files [file gkad068_supplemental_files.zip › Movie S1 legend.pdf]

Movie S1. Search for the chromosomal integration site *attB* by ICE*Bs1.* Time lapse fluorescence microscopy of transconjugant cells following mating of MMB1277 as a donor and SAM318 as a recipient. ICE*Bs1* was labelled with GFP (green dot, bottom left); *attB* was labelled with mApple2 (red dots, bottom right); phase contrast images (top right); overlay of GFP (ICE*Bs1*), mApple2 (*attB*) and phase contrast images (top left).
