# Supplementary material for: Target search by an imported conjugative DNA element for a unique integration site along a bacterial chromosome during horizontal gene transfer
Source: Nucleic Acids Res. 2023 Feb 10;51(7):3116–29. doi: 10.1093/nar/gkad068 (PMC10123120; doi:10.1093/nar/gkad068)
Supplement: gkad068_Supplemental_Files [file gkad068_supplemental_files.zip › Supplementary Data.pdf]

Target search by an imported conjugative DNA element for a unique integration site along a bacterial chromosome during horizontal gene transfer

Supplementary Data

Rinat Arbel-Goren[1#*], Saria A. McKeithen-Mead[2#], Dominik Voglmaier[1], Idana Afremov[1], Gianluca Teza[1], Alan D. Grossman[2,*], Joel Stavans[1,*]

[1] Department of Physics of Complex Systems
Weizmann Institute of Science
Rehovot 76100
Israel

[2] Department of Biology
Massachusetts Institute of Technology
Cambridge, MA 02139
USA

[#]Equal contributors

* **To whom correspondence should be addressed:** rinat.goren@weizmann.ac.il
joel.stavans@weizmann.ac.il
adg@mit.edu

In this supplementary information we provide Figures S1-S6 to support the discussion in the main text. In what follows, the notations and abbreviations are the same as in the main text and the equations and figures refer to those therein

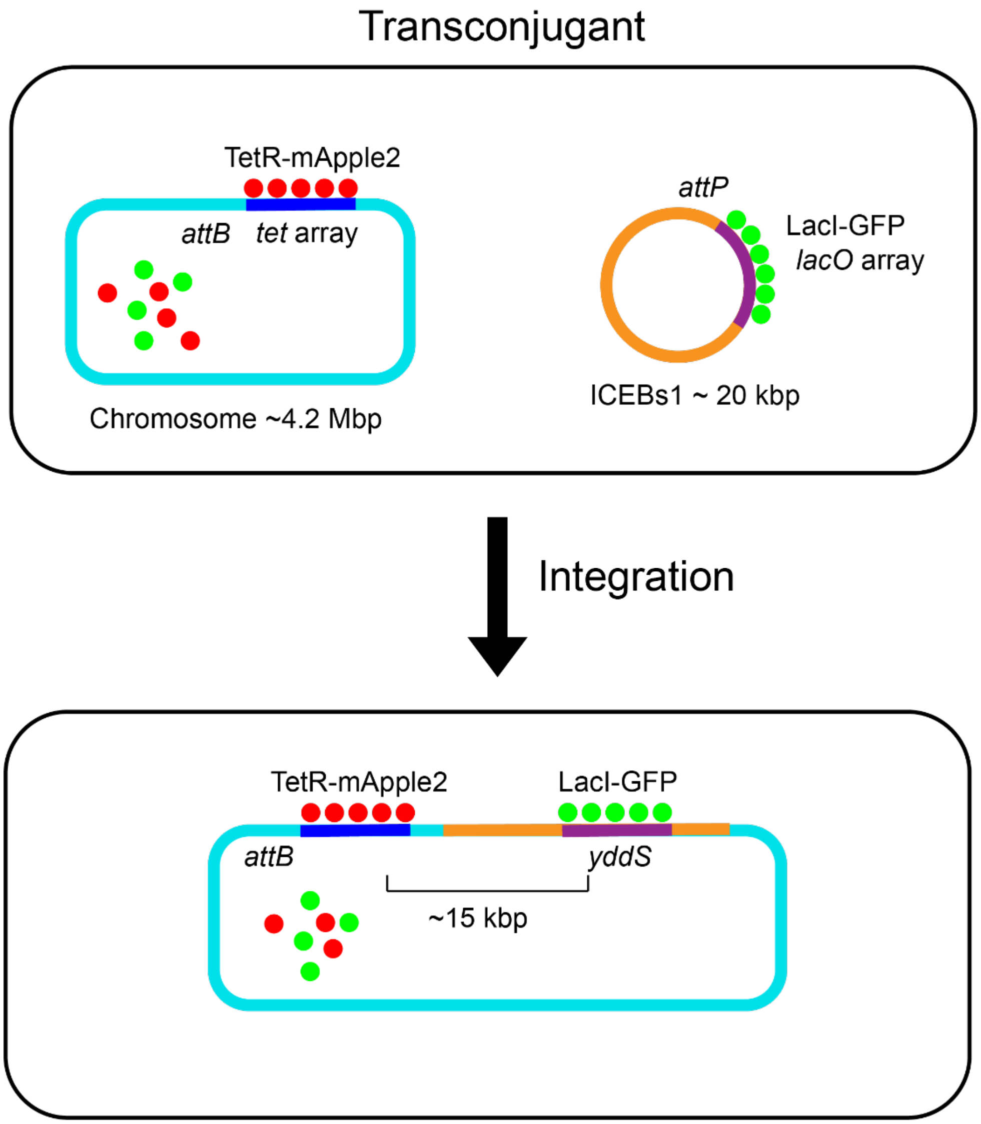

Figure S1. Experimental scheme. Top: transconjugant cell depicting an ICE*Bs1* (orange) labelled with LacI-GFP fusion (green circles) bound to a *lacO* array (violet) prior to integration into the transconjugant's chromosome (cyan). The integration site in the chromosome (*attB*) is labelled with a TetR-mCherry fusion (red circles) bound to a *tetO* array. After integration, both labelled arrays are separated by a contour distance of ~15kbp.

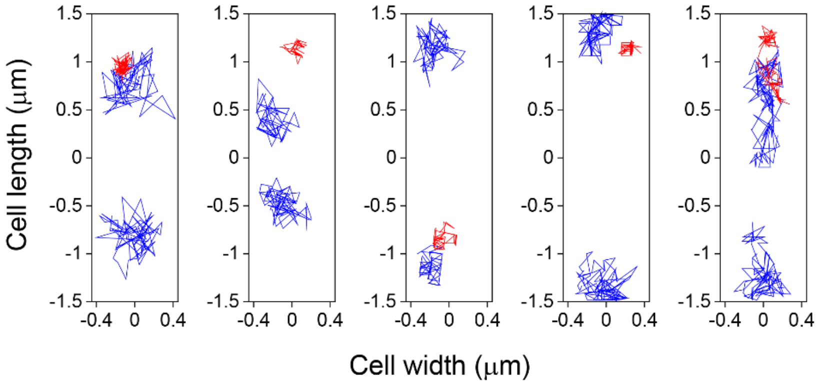

Figure S2. Kinetically trapped states of ICE*Bs1*. Two-dimensional trajectories of *attB* (blue) and ICE*Bs1* (red) in five representative transconjugants. The interval between successive frames was ~4 sec.

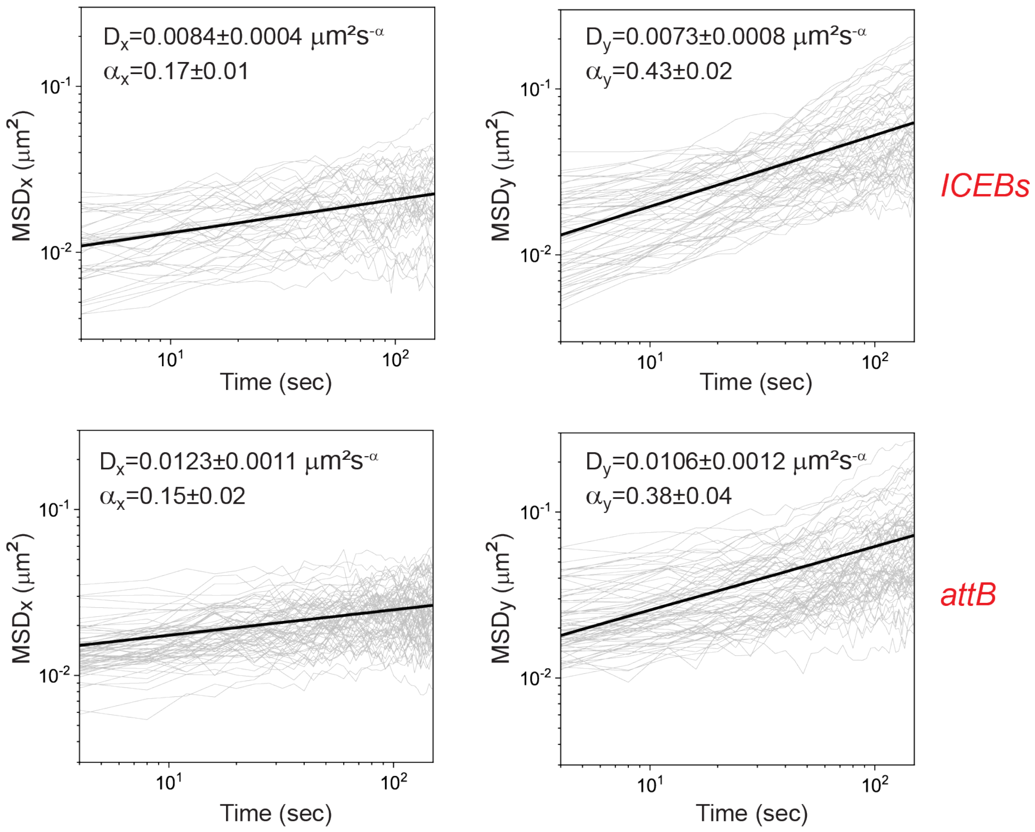

Figure S3. Mean square displacements of integrated ICE*Bs1* and *attB* are similar. Top: Mean squared displacements MSD in the transversal (left) and longitudinal (right) of individual ICE*Bs1*s trajectories (MSD_x and MSD_y respectively) in cells in which ICE*Bs1* is integrated and cannot excise (SAM837). Bottom: MSD_x and MSD_y of *attB*. Traces corresponding to individual trajectories are in grey while black lines are power law fits to the mean of the individual traces in each panel.

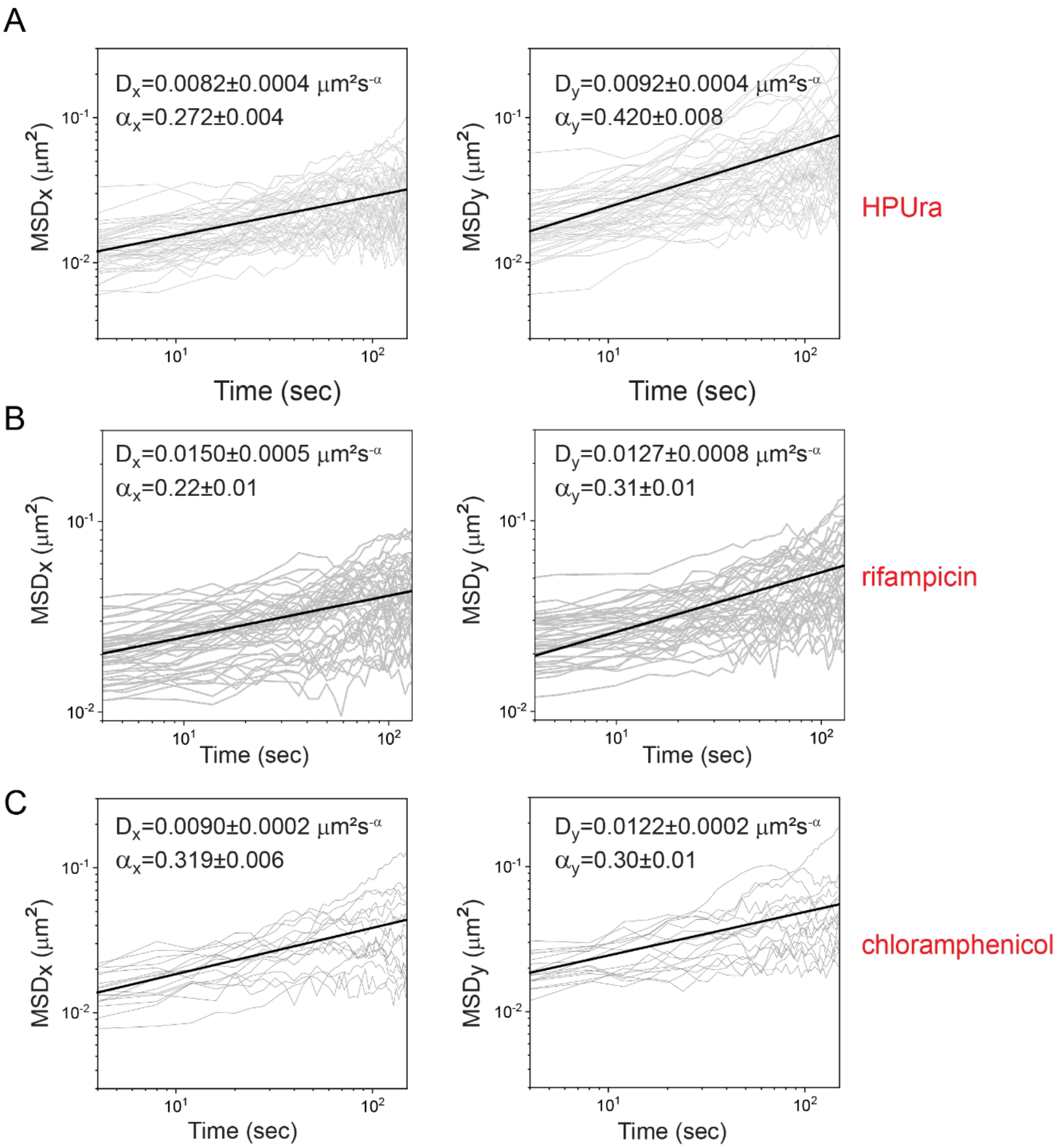

Figure S4. Effects of inhibitors of DNA replication, transcription, and translation on the dynamics of *attB*. Mean squared displacements MSD_x (left) and MSD_y (right) directions of *attB* in transconjugants treated with: (**A**) HPUra to inhibit DNA replication; (**B**) rifampicin to inhibit transcription; and (**C**) chloramphenicol to inhibit translation.

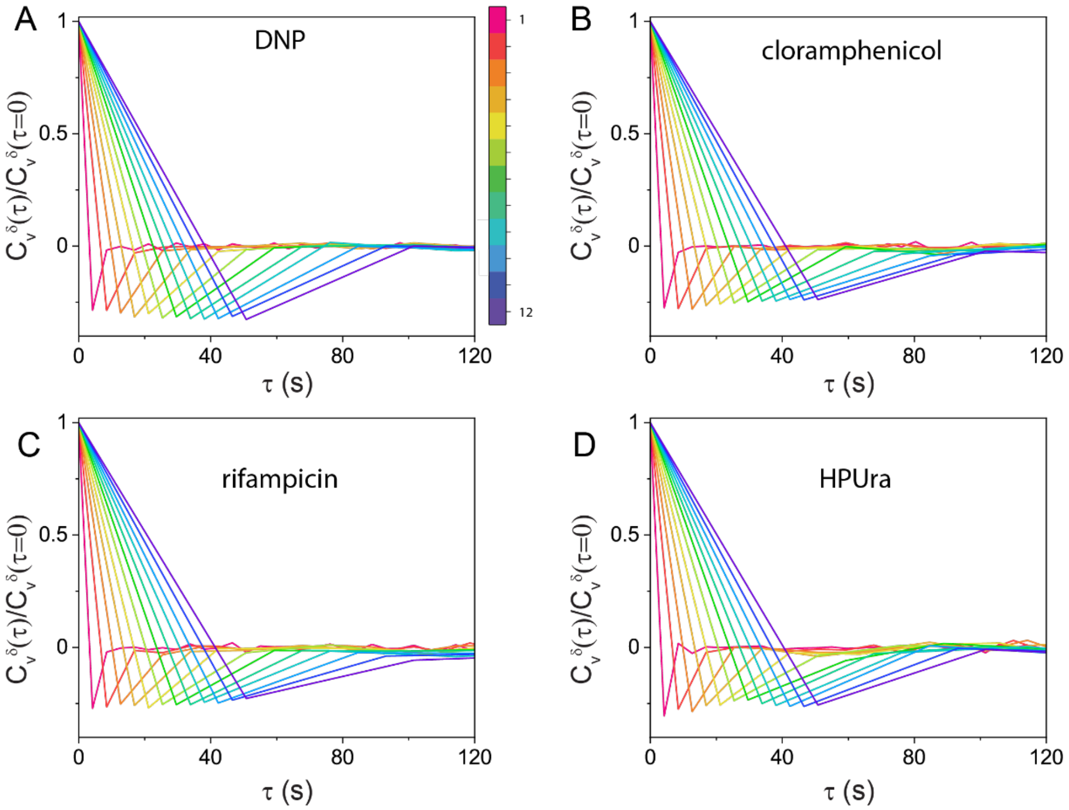

Figure S5. Velocity autocorrelation functions of *attB* trajectories in transconjugants under various treatments. Normalized velocity autocorrelation function $C_u^\delta(\tau)/C_u^\delta(0)$ of *attB* trajectories as a function of time, for different lag times δ, which are multiples of the interval between successive frames (4.23 sec), in cells treated with (**A**) DNP, (**B**) the translation inhibitor chloramphenicol, (**C**) the transcription inhibitor rifampicin and (**D**) the DNA replication inhibitor HPUra. The velocity $\bar{u}$ between positions separated by intervals δ ranging from 4 to 40 sec as indicated by the color scheme bar. The color scheme represents the values of δ from small (red) to large (purple).

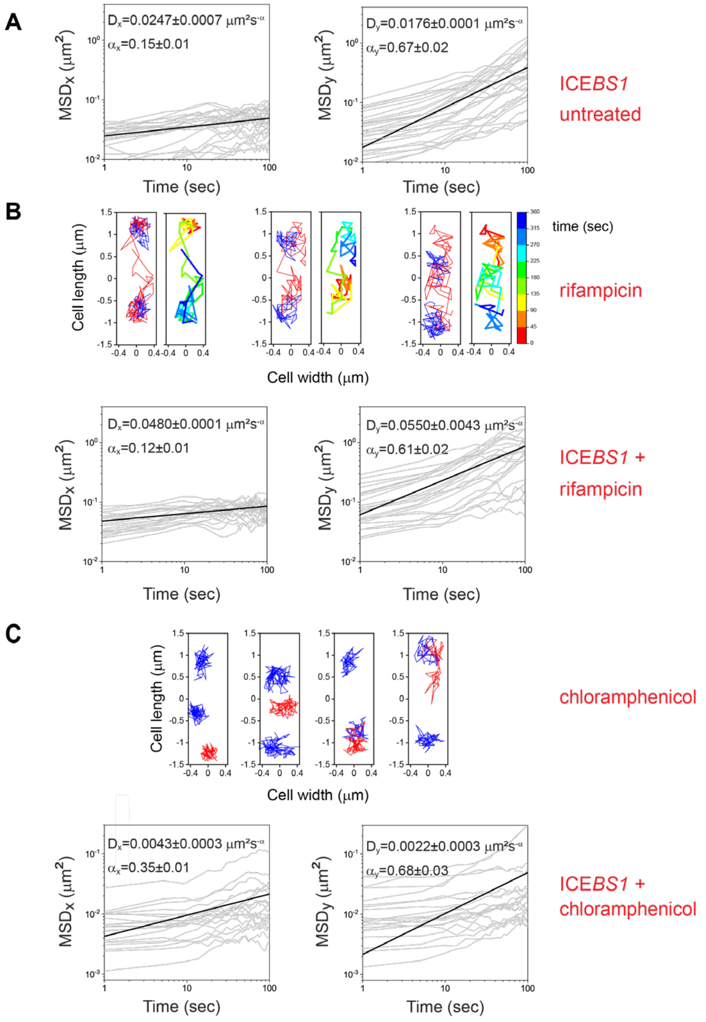

Figure S6. Effects of perturbations to chromosomal structure on the dynamics of ICE*Bs1*. (**A**) Fluorescence images of integrated ICE*Bs1* (green), *attB* (red) and the overlay (strain SAM837). (**B**) Effects of rifampicin treatment on the dynamics of ICE*Bs1*. Top panels show examples of two-dimensional trajectories of *attB*(blue) and ICE*Bs1* (red) in three typical cells, together with the same ICE*Bs1* trajectories in the right, color coded by time (rightmost panel). Bottom panels: mean squared displacement in the transversal (MSD_x, left panel) and longitudinal (MSD_y, right panel) directions of individual two-dimensional ICE*Bs1* trajectories (grey). The mean MSD in each case is shown by a black line and its fit by a power $MSD = Dt^{\alpha}$ is shown in red. The corresponding values of the effective diffusion coefficient D and exponent α are given in each panel. (**C**) Same as in (B) but in cells treated with chloramphenicol.
